# Doxing: What Adolescents Look for and Their Intentions

**DOI:** 10.3390/ijerph16020218

**Published:** 2019-01-14

**Authors:** Mengtong Chen, Anne Shann Yue Cheung, Ko Ling Chan

**Affiliations:** 1 Department of Applied Social Sciences, The Hong Kong Polytechnic University, Hong Kong, China; jenna.mt.chen@polyu.edu.hk; 2 Faculty of Law, The University of Hong Kong, Hong Kong, China; anne.cheung@hku.hk

**Keywords:** doxing, cyberbullying, intentions, perpetration, victimization, risk factors

## Abstract

Doxing is a form of cyberbullying in which personal information on others is sought and released, thereby violating their privacy and facilitating further harassment. This study examined adolescents’ doxing participation using a representative sample of 2120 Hong Kong secondary school students. Just over one in 10 had engaged in doxing, and doxing behavior significantly increased the probability of disclosing personal information on others (odds ratio ranged between 2.705 and 5.181). Social and hostile doxing were the two most common forms of doxing. Girls were significantly more likely to conduct social doxing (χ^2^ = 11.84, *p* < 0.001), where their target was to obtain social information (χ^2^ = 4.79, *p* = 0.029), whereas boys were more likely to engage in hostile doxing aimed at obtaining personally identifiable information (χ^2^ = 4.31, *p* = 0.038) and information on others’ current living situations (χ^2^ = 4.17, *p* = 0.041). Students who had perpetrated doxing acts were more likely to have experienced information disclosure as victims, perpetrators, or bystanders. Future studies should examine doxing’s impacts and its relationship with other forms of cyberbullying and traditional bullying. Because doxing may lead to on- and off-line harassment, family, adolescents, schools, and communities must work together to develop effective approaches for combating it.

## 1. Introduction

Cyberbullying victimization and perpetration among adolescents constitute a growing public health concern [1,2]. The intentions, risk factors, and consequences of cyberbullying are widely recognized by scholars [3,4]. The prevalence estimates for cyberbullying victimization among youths range from 10–40% across studies using different definitions, samples, and sampling methods [5]. Cyberbullying in adolescents typically refers to situations in which one adolescent is intentionally harassed by another adolescent or group of adolescents through the use of technology [6], and it can take numerous forms, including flaming (i.e., the posting of provocative or offensive messages), harassment, exclusion, cyberstalking, impersonation, outing and trickery, and sexting [7]. It is also perpetrated on a variety of electronic platforms, including social networking sites (SNSs), instant messenger services, search engines, e-mail, chat rooms, websites, and forums.

Risk factors associated with cyberbullying include adolescents’ behavior problems, low prosocial behavior, hyperactivity, perceived difficulties, feeling insecure at school, higher level of traditional victimization, etc. [8,9]. Protective factors for cyberbullying include family social support and indulgent parenting [10,11]. Meta-analyses and review studies have demonstrated cyberbullying victimization to be associated with mental and physical health problems in adolescents, including stress, depression, anxiety, low self-esteem, loneliness, addictive behaviors, somatic symptoms, and suicidal ideation [1,5]. In addition, adolescents with cyberbullying perpetration or victimization experiences also tend to be involved in traditional bullying [8,12]. Cyberbullying can be a component or extension of traditional bullying [13], and we argue that cyberbullying victimization can also increase the risk of being bullied in real life.

One form of cyberbullying is doxing, which refers to obtaining and disclosing the personal information of others without their consent. Not only does doxing violate victims’ information privacy [7], but it can also facilitate harassment against them in cyberspace and even lead to traditional bullying and violence because their personally identifiable information and physical location are often made public. Thus, doxing links harassment in cyberspace to harassment in real life. It also intensifies the power imbalance between perpetrators and victims—perpetrators remain anonymous, whereas victims are exposed to public scrutiny and become more accessible, both in cyberspace and the physical world.

In the study reported herein, we examined this particularly egregious form of cyberbullying in adolescents, which has drawn increasing attention in recent years. Our purpose was to obtain a preliminary understanding of doxing in this vulnerable population, including its prevalence, perpetrators, intentions, and association with information disclosure to provide researchers and practitioners with directions for future research and attention.

### 1.1. Definition of Doxing

The term “doxing” originates from the abbreviation “docs”, which simply stands for “documents” [14]. According to the Cambridge Dictionary [15], doxing means “searching for or publishing private or identifying information on a particular individual on the Internet without their permission”. Perpetrators typically engage in doxing with the malicious intent to humiliate, threaten, intimidate, or punish a particular individual [16]. Even if the person conducting the doxing is not hostile, disclosing private personal information on others can cause serious harm. In this paper, we adopt a broader concept of doxing that distinguishes the different intentions for engaging in it.

Doxing is different from outing and trickery in Willard’s definition of cyberbullying [7]. One critical feature of doxing is the process of searching for information on others via the Internet, instead of tricking the victims into communication or disclosing embarrassing information. In addition, doxing does not necessarily have to search for or disclose embarrassing information about the victims [16]. Target information of doxing can be any personal, private, or sensitive information. The conduct of searching for information on others also differentiates doxing from simply disclosing information.

Doxing is unsophisticated at the technical level [17]. Basic information on others can be gathered using publicly available sources, such as SNSs and online forums. When a perpetrator experiences difficulty accessing certain information, he or she can often deduce it from a limited set of initial information, such as a person’s e-mail address and phone number [14]. Doxing can happen to anyone—celebrities and ordinary people alike—and its targets include children and adolescents [16]. Doxing’s victims are individuals of interest to its perpetrators, whether they like or dislike them. By disclosing victims’ personal information, doxing perpetrators encourage others to participate in online harassment. Although online privacy is an important issue for all Internet users, there is a lack of awareness of and policy approaches toward doxing.

### 1.2. Online Self-Disclosure and Doxing in Adolescents

Today’s adolescents have been born into societies where Internet technology is an indispensable part of life. Information and Communications Technology (ICT) has changed social interactions, including the way in which we acquire information, express our views, and communicate with others. More than 80% of adolescents in the United States and European Union have their own personal social networking pages [18]; approximately 90% of adolescents in Hong Kong have at least one social networking account [19]; and 90.9% of adolescents in mainland China use an instant messenger, with 51.6% having a Weibo account, a Chinese version of Twitter [20].

As an important part of the social environment, the cyber environment affects and shapes adolescents’ psychosocial development. Adolescents use SNSs as their primary way of communicating with others, including friends and peers, and even strangers [21]. Sharing one’s own personal information on SNSs is also a growing trend among adolescents. By widely sharing such information and expressing themselves online, adolescents establish their self-identity and form friendships and peer relationships. However, managing information online is a more complex process than simply posting it, particularly for adolescents, who often find it difficult to determine the boundaries of appropriate self-disclosure for different groups of people [22]. Many adolescents share such personally identifiable information as their full name, sex, birthday, school, relationship status, and e-mail address, as well as personal photos and videos [23]. Therefore, online self-disclosure is a long-standing concern for parents, schools, and society.

In addition to engaging in online self-disclosure, adolescents can also easily access information on others using SNSs. The result is that adolescents have little control over the potential use of their personal information by others when they encounter doxing [24]. The increasing misuse of personal information online (e.g., cyber-harassment, cyberstalking) means that sharing it can be very risky [25]. Adolescents’ lack of concern over online privacy can even result in real-life violence in the form of intimidation, humiliation, physical attacks, and kidnapping [23]. The widespread accessibility of Internet technology via smartphones and laptops poses a considerable challenge for parents and schools in supervising the online self-disclosure behavior of adolescents and protecting them from cyber violence.

### 1.3. Research Gaps: Lack of Empirical Studies on Doxing

Like other forms of cyberbullying, doxing can exert negative impacts on adolescents. Understanding the phenomenon in the adolescent context is important to understanding the connection between doxing and other forms of cyberbullying. Because doxing among adolescents has emerged as a public health concern, we require research on its prevalence in that population, as well as on the reasons that adolescents engage in doxing and the factors that increase or decrease the likelihood of an adolescent becoming a victim or perpetrator.

Dozens of studies have demonstrated the high prevalence of cyberbullying among adolescents, but only a few have included doxing in operationalizing cyberbullying, for example [5]. In addition, the extant research on doxing is primarily qualitative or based on data collected from online text-sharing sites and SNSs, resulting in a lack of quantitative evidence [17]. Therefore, there is a need to estimate doxing prevalence among adolescents using evidence from well-designed, population-based surveys.

Several researchers have discussed the factors influencing adolescents’ disclosure of their own personal information on SNSs [23,25], but few have examined doxing targeted at adolescents, which may be the result of the ubiquity of online self-disclosure in that population and has potentially serious consequences in both cyberspace and the real world. Therefore, it is important for researchers to examine adolescents’ participation in doxing and in exposing others’ data.

### 1.4. The Current Study

As noted, there are growing concerns about adolescents’ disclosure of personal information on others online and consequential cyberbullying against them. The aim of the current study was to obtain a preliminary understanding of doxing among adolescents. We conducted a quantitative survey examining doxing perpetration among a representative sample of secondary school students in Hong Kong. Our main research objective and hypotheses were as follows.

Objective 1: To examine the prevalence of doxing perpetration among adolescents.

Objective 2: To examine whether doxing influences the risk of disclosing personal information on others.

- Hypothesis 2.1: Adolescents’ doxing behavior increases the probability of disclosing personal information on others.

Objective 3: To examine adolescents’ intentions for engaging in doxing.

- Hypothesis 3.1: Adolescents’ intentions for engaging in doxing differs by gender.

Objective 4: To examine adolescents’ target information of doxing.

- Hypothesis 4.1: Adolescents’ target information of doxing differs by gender.
- Hypothesis 4.2: Adolescents’ target information of doxing differs by the target persons.

Objective 5: To examine factors that are associated with adolescents’ perpetration of doxing.

- Hypothesis 5.1: Adolescents’ perpetration of doxing is associated with particular demographic characteristics.
- Hypothesis 5.2: Adolescents’ perpetration of doxing is associated with experiences of information disclosure perpetration and victimization.

## 2. Methods

### 2.1. Study Design and Sample

A school-based cross-sectional survey was carried out in Hong Kong in 2018. The target respondents were Secondary 2 (S2) to Secondary 5 (S5) students, and the age of students mainly ranged from 13 to 17 years. To recruit students of different socioeconomic backgrounds, we randomly selected 22 government schools, aided schools, direct subsidy scheme, and private schools from three regions of Hong Kong, including Hong Kong Island, Kowloon, and the New Territories. One class from each grade was then randomly sampled. The final survey sample totaled 2120 respondents, including 1123 boys and 997 girls. Table 1 presents the respondents’ demographic characteristics. Their mean age was 15.11 (*SD* = 1.45), with no significant difference between boys and girls. The numbers of students in the various grades were also comparable. Though more than one fourth of students did not know the educational level of their parents, the majority of the students were from parents with Upper Secondary educational level or above.

### 2.2. Procedures

The participating students were asked to fill out a self-administrated questionnaire in a classroom setting after obtaining their informed written consent. Research assistants were on hand to provide assistance to the students in completing the questionnaire where necessary. Ethical approval for the survey was obtained from the Human Research Ethics Committee of the University of Hong Kong (EA1602044) prior to study commencement.

### 2.3. Measurements

#### 2.3.1. Demographic Characteristics

The questionnaire collected demographic information on the participants, including their age, sex, year of secondary school, and their father’s and mother’s educational level.

#### 2.3.2. Doxing Experience

The definition and examples of doxing were provided first to let participants establish a basic understanding of doxing. A single questionnaire item—“Have you ever conducted doxing on the Internet?”—was then used to identify respondents with doxing perpetration experience. Those who answered in the affirmative also answered questions on the platform on which they have engaged in doxing, with the platform options including, but not limited to, e-mail, chatrooms, forums, instant messengers, search engines, SNSs, blogs, web pages, and video-sharing websites. Finally, we asked students to select their doxing targets and indicate whether they liked or disliked them.

#### 2.3.3. Target Information of Doxing

The respondents who admitted to doxing were then asked to indicate the personal information they had found on their targets, such as their name, birthday, ID card number, phone number, and the like. A total of 28 items were included and categorized into seven types, as shown below:

(1) Name;
(2) Social information, including four items: birthday, school name, relationship status, and personal photos or videos. Items in Category 1 and 2 are often publicly accessible, being posted on personal social networking pages.
(3) Personally identifiable information, including four items: ID card number, passport number, bank account number, and usernames/passwords of online accounts;
(4) Current living situation, including four items: home telephone number, home address, locations, and parents’ names;
(5) Education information, including two items: student card and academic performance;
(6) Private information, including seven items: cell phone number, personal e-mail address, odd habits, intimate photos or videos, obscene/indecent photos or videos, embarrassing photos or videos, and private Internet or text conversations;
(7) Sensitive information, including six items: sexual orientation, sexual life, racial or ethnic origin, political opinions, religious beliefs, and medical records, whose disclosure is prohibited by the European Union (EU) General Data Protection Regulation [26].

#### 2.3.4. Disclosing Information on Others

The research team developed a self-constructed questionnaire with 28 items measuring adolescents’ experience of disclosing personal information on others on the Internet without their consent. The items are the same as those asked above in the Target Information of Doxing, including name, birthday, ID card number, phone number, and the like. Each item was rated on a six-point scale, ranging from “0 = never” to “5 = over 15 times.” The measure demonstrated a very high degree of internal consistency (Cronbach’s *α* = 0.995), indicating strong intercorrelation among the items. Such intercorrelation is acceptable because doxing involves the disclosure of multiple private details on individuals, or even full text files containing their personal information. The scale was also divided into subscales corresponding to the aforementioned categories of target information. We then recoded the items into single dichotomous variables, with a score of 1 indicating that the respondent had disclosed another’s personal information at least once, and a score of 0 meaning that he or she had never engaged in such disclosure. Respondents with a score of 1 for at least one item were coded as perpetrators who “had disclosed personal information on others” within a particular subscale or on the overall scale.

#### 2.3.5. Doxing Victimization Experience

We also identified the victims of doxing by asking the participating students about their response when they discovered that their personal information had been disclosed. We categorized students who had done nothing because they were not bothered by the disclosure or did not regard it as a problem as non-victims. The students who thought they were affected, tried to solve the problem, or asked for help were categorized as victims.

#### 2.3.6. Information Disclosure Bystanders

We used a questionnaire which contained the same 28 items of personal information to determine whether the participating adolescents had witnessed others posting personal information of third parties on the Internet, with the items rated on the same six-point scale ranging from “0 = never” to “5 = over 15 times”. The items were recoded into single dichotomous variables, with a score of 1 indicating that the respondent had witnessed doxing by others at least once, and a score of 0 meaning that he or she had never witnessed such information disclosure. Those who responded that they had observed such behavior at least once were coded as bystanders.

### 2.4. Statistical Analysis

To analyze the respondents’ demographic information and doxing experiences, we first performed descriptive analyses and chi-square tests. We then conducted logistic regression analyses to examine the association between adolescents’ doxing behavior and their disclosure of personal information on others. An odds ratio (OR) >1 indicates an increased probability of disclosing personal information. We estimated that for adolescents who conducted doxing, the odds of releasing information about others increased. Finally, to identify the factors related to such behavior, including whether the doxing targets were people they liked or disliked, we carried out binary logistic regression analyses.

## 3. Results

### 3.1. Demographic Characteristics and Prevalence of Doxing

The distribution of demographic characteristics by gender in the study sample is presented in Table 1. In addition, Table 1 also presents the demographic characteristics of two groups of respondents: those who had conducted doxing and those who had not. A total of 259 students (12%) acknowledged having engaged in doxing. These students tended to be younger than those who had not engaged in such behavior (*t* = −2.32, *p* = 0.02), and, accordingly, were also in lower grades of secondary school (χ^2^ = 8.04, *p* = 0.045). Interestingly, significantly more girls than boys reported having conducted doxing (χ^2^ = 8.7, *p* = 0.003). 

### 3.2. Association between Doxing and Disclosure of Others’ Personal Information

As shown in Table 2, we found the participating adolescents’ doxing behavior to be strongly associated with whether they had disclosed personal information on others. As the results of searching for information on others, the odds of an adolescent who conducted doxing disclosing others’ personal information were much higher than those who did not conduct doxing. Increased odds were found with respect to the types of personal information often posted on personal networking pages, such as names (OR = 3.286), social information (OR = 3.438), and education information (OR = 2.946). Doxing was even found to be associated with the disclosure of personal information that should not be exposed, including personally identifiable information (OR = 4.16), private information (OR = 2.705), and sensitive information (OR = 5.181). Therefore, we confirmed Hypothesis 2.1 that adolescents’ doxing behavior increased the probability of disclosing personal information on others.

### 3.3. Intentions of Doxing

The adolescents who perpetrated doxing did so with different intentions. As shown in Table 3, 53.2% of adolescents admitted doxing people they liked, with girls (62%) more likely to do so than boys (41.2%; χ^2^ = 11.84, *p* < 0.001). This finding is consistent with the finding in Table 4 that the doxing girls (96.3%) were significantly more interested than their male counterparts (88.8%) in social data and trying to ascertain an individual’s relationship status and obtain his or her personal photos or videos (χ^2^ = 4.79, *p* = 0.029). In addition, more girls (86.7%) than boys (66.8%) chose SNSs as their doxing platform (χ^2^ = 13.59, *p* < 0.001). For social doxing, we can confirm Hypothesis 3.1 that adolescents’ intentions for engaging in doxing differed by gender. In sum, we found girls more likely than boys to engage in social doxing, which may be part of their social interactions.

### 3.4. Target Information of Doxing

Half of the doxing perpetrators chose targets whom they disliked. As shown in Table 4, significantly more boys than girls reported having obtained personally identifiable information (16.3%; e.g., ID card numbers, passport numbers; χ^2^ = 4.31, *p* < 0.05) and information on the current living situation (49.9%; e.g., home address, parents’ names; χ^2^ = 4.17, *p* < 0.05) of their victims through doxing. In addition, those who had only targeted individuals they disliked were more interested in obtaining personally identifiable information (22.1%; χ^2^ = 16.25, *p* < 0.001), current living situation information (58.1%; χ^2^ = 14.27, *p* = 0.003), and private information (77.3%; χ^2^ = 10.61, *p* = 0.014). Based on the results, we can confirm Hypotheses 4.1 and 4.2 that adolescents’ target information of doxing differed by gender and the targeted persons, with regard to personally identifiable information and information on students’ current living situation. Because private and sensitive information can be used to humiliate, harass, or attack individuals in both cyberspace and the real world, it can be concluded that boys are more likely to conduct hostile doxing than girls. In addition, the boys in this study were more inclined to use search engines, video-sharing websites, and forums for doxing, allowing them to obtain more information than would be possible using SNSs. The implication is that some boys engage in doxing with strong hostile intentions. 

### 3.5. Factors Associated with Adolescent Doxing

Table 5 lists the factors associated with adolescents’ doxing behavior toward individuals they like and dislike. Boys were found to be less likely than girls to dox those they like (OR = 0.417, *p* < 0.001). However, neither age nor educational level was a predictor of doxing behavior. Doxing behavior against liked targets was associated with being a perpetrator of personal information disclosure (OR = 1.942, *p* = 0.042). Doxing behavior against disliked targets was associated with being both a perpetrator and victim (OR = 2.556, *p* = 0.005), and being a bystander (OR = 1.746, *p* = 0.038). Based on results of logistic regressions, we confirmed Hypotheses 5.1 and 5.2 that adolescents’ perpetration of doxing was associated with gender, and their experiences of information disclosure perpetration and victimization.

## 4. Discussion

The findings of this quantitative study have promoted the understanding of doxing among adolescents. Just over one in 10 of the secondary school students we surveyed had previously engaged in doxing, and those who had done so were more likely to be girls, younger in age, and in a lower secondary school grade. A prior study found young people in Hong Kong to display higher levels of trust in social media and to be more likely to disclose sensitive personal information such as health-related information with others on SNSs than their U.S. and Korean counterparts [27], which may explain the prevalence of doxing perpetration among the Hong Kong adolescents in our study. The disclosure of personal information online increases one’s likelihood of experiencing cyber-harassment, and SNS use can intensify doxing perpetration among adolescents, as demonstrated by approximately 80% of the students who reported doxing in our study stating that they had engaged in such behavior on SNSs.

We also found that adolescents who conducted doxing had greater odds of disclosing others’ personal information. Adolescents’ disclosing personal information on others can be implied by their searching for and obtaining information on others via the Internet. Because publicly exposing an individual’s personal information leaves him or her vulnerable to harassment, doxing can encourage others to engage in cyberbullying. Moreover, the disclosure of personally identifiable information and physical location information can even result in real-life harassment and attacks. Our findings thus highlight the need for greater attention to be paid to doxing among adolescents.

Although all doxing can result in negative consequences, it should be noted that adolescents who conduct doxing do so with different intentions. The current study thus constitutes a preliminary investigation of adolescents’ reasons for engaging in doxing. Based on its findings, we categorized their intentions into two general types: social doxing and hostile doxing. Half of the respondents who admitted doxing did so in part to fulfill their social needs, with their doxing targeted primarily at obtaining social data, such as names and relationship status, and personal photos and videos. Half of the doxing perpetrators in this study were revealed to target people they dislike, probably with the malicious intention of harassing or attacking them.

In addition to social information, which are usually posted on SNSs, these respondents also targeted their victims’ personally identifiable information, information on their living situation, and private and sensitive information. The disclosure of such information can be very risky, as it can facilitate further cyberbullying and even real-life violence against victims. Both boys and girls engage in doxing, although we found the latter to be significantly more likely to conduct social doxing via SNSs. Girls were found to have more social anxiety than boys in previous research [28,29], which may explain their intentions of social doxing. Even though we identified no gender differences in hostile doxing, significantly more boys than girls targeted personally identifiable and physical location information and other types of private and sensitive data. Research on cyberbullying has also reported inconsistent findings about the rates of cyberbullying perpetration by gender. Some research found no significant difference between girls and boys, whereas some research found that boys were more likely to engage in cyberbullying as perpetrators [5].

Our results also revealed that the students who had conducted doxing had also experienced information disclosure as victims, perpetrators, or bystanders, which is consistent with prior research on cyberbullying among adolescents showing that previous cyberbullying experience is a predictor of perpetration [30,31]. Doxing behavior in adolescents may be initiated by victimization experience. Also, perpetrators’ own personal information may be exposed as revenge for their doxing behavior. Doxing can even encourage adolescents to participate in this form of cyberbullying as bystanders.

### 4.1. Limitations and Implications for Future Research

The present study had several limitations, which should be considered when interpreting its results. Firstly, because we used a self-constructed questionnaire to examine adolescents’ participation in doxing by investigating the different kinds of personal information they obtain from doxing, the questionnaire omitted potentially relevant items, such as victims’ previous traumatic experiences and mental health. Therefore, we suggest that future research should collect more in-depth information on adolescents’ doxing behavior.

Secondly, because we collected only the participating students’ basic demographic characteristics, including such family environmental factors as parental characteristics and family income and such individual psychosocial factors as addiction behavior and psychological well-being were not taken into account. A fruitful direction for further study would be to examine whether these factors increase or decrease the likelihood of an adolescent conducting doxing. Furthermore, as victimization within the family environment is associated with cybervictimization in adolescents, the association between doxing and family victimization also needs to be addressed [32].

Finally, as with other forms of cyberbullying, doxing can have serious consequences for victims, such as depressive symptoms, anxiety, interpersonal issues, and school refusal [3,33]. As the findings of the current study imply that doxing can also increase the risk of bullying and harassment in both cyberspace and real life, we recommend that future studies focus on the impacts of doxing and investigate its relationship with other forms of cyberbullying and traditional bullying.

### 4.2. Implications for Practice

Adolescents who have conducted doxing are more willing than those who have not to disclose personal information on others via SNSs, regardless of whether they like or dislike them. The nature and design of SNSs facilitate information disclosure, and the recipients of the disclosed information can be anyone. In addition, it appears that doxing perpetrators are sometimes also doxing victims, and we know that such victims are vulnerable to future cyber-harassment and cyberbullying. It is thus important to address the doxing problem among adolescents.

Doxing is not a homogeneous phenomenon: it is perpetrated with different intentions, and there are gender differences in the way it is perpetrated. Accordingly, multidisciplinary approaches encompassing the legal, education, social work, and technology arenas are needed to combat doxing. Firstly, it is important to inform adolescents of information privacy laws prohibiting the unauthorized disclosure of personal information, as well as the potentially serious consequences of hostile doxing and severe penalties for doxing [34]. Secondly, schools and parents need to provide adolescents with guidelines on online behavior. It is recommended that empathy education and training be included in intervention programs, which has been shown to be effective in preventing bullying [35]. For example, adolescents, particularly those who engage in hostile doxing, could be helped to view the doxing experience from the victims’ perspective and understand their feelings [3]. Thirdly, it is important to improve parent–adolescent relationships and parental involvement to reduce the likelihood of doxing [36]. Also, improving parenting practices can be a protective factor for adolescents’ doxing behavior [37]. Parental mediation strategies have been shown to be effective in reducing risky online behaviors in adolescents, and parent education programs that foster children’s internalization of social norms are well-received [23]. Finally, anti-abuse services that inform individuals when their personal information has been shared in a dox file have been proposed [17], and we further recommend that families, adolescents, schools, and communities work cooperatively to combat the problem of doxing.

## 5. Conclusions

More than one out of every 10 secondary school students has engaged in doxing, and doxing behavior is significantly associated with the disclosure of personal information on others. This paper contributes to our understanding of doxing by revealing the different doxing intentions and targets of adolescents. Because doxing increases the risk of information disclosure, consequently leading to the risk of harassment and attacks in both cyberspace and the physical world, it is important to develop effective approaches for combating the problem.

## Figures and Tables

**Table 1:** Demographic characteristics of participants.

Demographic Characteristics	Sex (%)				Have You Ever Conducted Doxing? (%)			
	Male (*n* = 1123; 53.0)	Female (*n* = 997; 47.0)	χ^2^	*p*	Yes (*n* = 259; 12.2)	No (*n* = 1861; 87.8)	χ^2^	*p*
Mean age (SD)	15.16 (1.44)	15.05 (1.46)	1.46	0.144	14.94 (1.36)	15.13 (1.46)	−2.32	0.02
Sex								
Male					9.8	90.2	8.70	0.003
Female					14.5	85.5		
Year of secondary school								
Secondary 2	24.7	25.2	0.40	0.941	25.2	24.9	8.04	0.045
Secondary 3	25.0	24.6			30.1	24.1		
Secondary 4	24.8	25.7			25.9	25.1		
Secondary 5	25.5	24.5			18.9	25.8		
Education level of father								
Don’t know	37.2	27.8	26.64	<0.001	29.0	33.2	6.75	0.24
No schooling/Pre-primary/Primary	6.2	5.7			5.0	6.1		
Lower Secondary (S.1–S.3)	14.5	19.1			14.3	17.0		
Upper Secondary/Sixth Form (S.4–S.7)	24.4	28.8			29.7	26.1		
Diploma/Certificate/ Sub-degree course	5.7	8.0			8.0	6.6		
Degree course or above	12.1	10.5			14.0	11.0		
Education level of mother								
Don’t know	35.4	24.4	35.24	<0.001	24.3	30.9	11.07	0.05
No schooling/Pre-primary/Primary	8.6	7.8			7.0	8.4		
Lower Secondary (S.1–S.3)	13.7	17.9			14.6	15.9		
Upper Secondary/Sixth Form (S.4–S.7)	25.5	31.5			31.1	28.0		
Diploma/Certificate/ Sub-degree course	7.5	9.1			10.8	7.9		
Degree course or above	9.3	9.3			12.2	8.9		

**Table 2:** Doxing as predictor of disclosure of others’ personal information.

Dependent Variable	B	OR	95% CI	*p*
Name	1.19	3.286	[2.491, 4.336]	<0.001
Social information	1.235	3.438	[2.634, 4.487]	<0.001
Personally identifiable information	1.425	4.16	[2.227, 7.77]	<0.001
Current living situation	1.237	3.445	[2.338, 5.075]	<0.001
Education information	1.08	2.946	[2.036, 4.262]	<0.001
Private information	0.995	2.705	[2.018, 3.625]	<0.001
Sensitive information	1.645	5.181	[3.352, 8.007]	<0.001

Note: B = coefficient; OR = odds ratio.

**Table 3:** Platforms and targets of doxing.

Information of Doxing	Male (*n* = 110; %)	Female (*n* = 149; %)	Total (*n* = 259; %)	χ^2^	*p*
Targets of doxing					
People whom you like	41.2	62	53.2	11.84	<0.001
People whom you dislike	57	45.9	50.7	2.05	0.152
Platform of doxing					
E-mail	11.4	2.4	6.2	6.47	0.011
Chat rooms	9.9	11.4	10.7	0.16	0.686
Forums	29.4	9.5	18.0	14.35	<0.001
Instant messengers	48.4	51.5	50.2	0.04	0.835
Search engines	43.0	23.8	31.9	11.46	<0.001
Social networking sites	66.8	86.7	78.2	13.59	<0.001
Blogs	7.2	3.2	4.9	1.63	0.202
Web pages	9.6	11.3	10.6	0.12	0.728
Video-sharing websites	24.3	13.3	18.0	4.63	0.031
Other	0.8	0.4	0.6	0.03	0.873

**Table 4:** Personal information on targets obtained through doxing (*n* = 259).

Doxed Information	Sex				Target of Doxing						
	Male (*n* = 110; %)	Female (*n* = 149; %)	χ ^2^	*p*	Someone I Like Only (*n* = 80; %)	Someone I Dislike Only (*n* = 70; %)	People I Like and Dislike (*n* = 59; %)	No Special Targets and Others (*n* = 50; %)	χ^2^	*p*	Total
Name	92.7	95.6	0.52	0.473	96.3	90.6	95.8	95.1	3.32	0.357	94.4
Social information	88.8	96.3	4.79	0.029	92.7	91.6	92.6	96.3	1.09	0.779	93.1
Personally identifiable information	14.6	6.3	4.31	0.038	3.1	22.1	9.1	3.7	16.25	<0.001	9.8
Current living situation	49.9	35.5	4.17	0.041	32.2	58.1	46.0	27	14.27	0.003	41.6
Education information	25.2	24.1	0.38	0.537	22.6	24.9	32.1	18.2	4.02	0.259	24.6
Private information	65.1	64.3	0.06	0.801	50.5	77.3	68.9	63.4	10.61	0.014	64.6
Sensitive information	39.5	35.2	0.11	0.743	34.8	40.4	45.8	25.7	4.659	0.199	37.1

**Table 5:** Logistic regression of factors associated with doxing (*n* = 259).

Associated Factors	Targets Whom They Like			Targets Whom They Dislike		
	*B*	OR [95% CI]	*p*	*B*	OR [95% CI]	*p*
Sex						
Male	−0.874	0.417 [0.253, 0.689]	< 0.001	0.359	1.432 [0.875, 2.342]	0.153
Female	1.000			1.000		
Age	−0.017	0.983 [0.821, 1.178]	0.856	0.008	1.008 [0.842, 1.207]	0.931
Year of secondary school						
Secondary 2	−0.087	0.917 [0.439, 1.916]	0.817	−0.254	0.776 [0.371, 1.623]	0.5
Secondary 3	0.288	1.333 [0.654, 2.717]	0.428	−0.215	0.807 [0.396, 1.643]	0.554
Secondary 4	0.348	1.417 [0.656, 3.06]	0.375	−0.167	0.846 [0.393, 1.821]	0.669
Secondary 5	1.000			1.000		
Disclosing information						
Victim only	0.093	1.098 [0.478, 2.521]	0.826	0.06	1.062 [0.459, 2.459]	0.888
Perpetrator only	0.664	1.942 [1.023, 3.685]	0.042	0.217	1.243 [0.661, 2.335]	0.499
Both	0.178	1.194 [0.637, 2.241]	0.58	0.938	2.556 [1.336, 4.889]	0.005
Bystander	0.289	1.335 [0.792, 2.249]	0.278	0.491558	1.746 [1.03, 2.96]	0.038

Note: B = coefficient; OR = odds ratio.

## References

[1] Selkie E.M., Fales J.L., Moreno M.A. (2016). Cyberbullying prevalence among US middle and high School–aged adolescents: A systematic review and quality assessment. J. Adolesc. Health.

[2] Wong D.S.W., Chan H.C., Cheng C.H.K. (2014). Cyberbullying perpetration and victimization among adolescents in Hong Kong. Child Youth Serv. Rev..

[3] Ang R.P. (2015). Adolescent cyberbullying: A review of characteristics, prevention and intervention strategies. Aggress. Violent Behav..

[4] Thomas H.J., Connor J.P., Scott J.G. (2015). Integrating traditional bullying and cyberbullying: Challenges of definition and measurement in adolescents―A review. Educ. Psychol. Rev..

[5] Kowalski R.M., Giumetti G.W., Schroeder A.N., Lattanner M.R. (2014). Bullying in the digital age: A critical review and meta-analysis of cyberbullying research among youth. Psychol. Bull..

[6] Hutson E. (2016). Cyberbullying in Adolescence. Adv. Nurs. Sci..

[7] Willard N.E. (2007). Cyberbullying and Cyberthreats: Responding to the Challenge of Online Social Aggression, Threats, and Distress.

[8] Sourander A., Klomek A.B., Ikonen M., Lindroos J., Luntamo T., Koskelainen M., Ristkari T., Helenius H. (2010). Psychosocial risk factors associated with cyberbullying among adolescents: A population-based study. Arch. Gen. Psychiatry.

[9] Cappadocia M.C., Craig W.M., Pepler D. (2013). Cyberbullying: Prevalence, Stability, and Risk Factors During Adolescence. Can. J. Sch. Psychol..

[10] Fanti K.A., Demetriou A.G., Hawa V.V. (2012). A longitudinal study of cyberbullying: Examining risk and protective factors. Eur. J. Dev. Psychol..

[11] Martínez I., Murgui S., Garcia O.F., Garcia F. (2019). Parenting in the digital era: Protective and risk parenting styles for traditional bullying and cyberbullying victimization. Comput. Hum. Behav..

[12] Chang F.-C., Lee C.-M., Chiu C.-H., Hsi W.-Y., Huang T.-F., Pan Y.-C. (2013). Relationships among cyberbullying, school bullying, and mental health in Taiwanese adolescents. J. Sch. Health.

[13] Olweus D. (2013). School bullying: Development and some important challenges. Annu. Rev. Clin. Psychol..

[14] Lee J. What is doxing and how does it affect your privacy. https://www.makeuseof.com/tag/what-is-doxing-and-how-does-it-affect-your-privacy-makeuseof-explains/.

[15] Cambridge Dictionary Doxing. https://dictionary.cambridge.org/dictionary/english/doxing.

[16] Douglas D.M. (2016). Doxing: A conceptual analysis. Ethics Inf. Technol..

[17] Snyder P., Doerfler P., Kanich C., McCoy D. Fifteen minutes of unwanted fame: Detecting and characterizing doxing. Proceedings of the 2017 Internet Measurement Conference.

[18] Walrave M., Poels K., Antheunis M.L., Van den Broeck E., van Noort G. (2018). Like or dislike? Adolescents’ responses to personalized social network site advertising. J. Mark. Commun..

[19] Public Opinion Programme of The University of Hong Kong Youth survey on usage of Internet and social network websites. https://www.hkupop.hku.hk/english/report/microsoft10/content/finding.html.

[20] China Internet Network Information Center A report on the Internet behavior of adolescents in China. http://www.cnnic.net.cn/hlwfzyj/hlwxzbg/qsnbg/201608/P020160812393489128332.pdf.

[21] Spies Shapiro L.A., Margolin G. (2014). Growing up wired: Social networking sites and adolescent psychosocial development. Clin. Child Fam. Psychol. Rev..

[22] Min J., Kim B. (2014). How are people enticed to disclose personal information despite privacy concerns in social network sites? The calculus between benefit and cost. J. Assoc. Inf. Sci. Technol..

[23] Liu C., Ang R.P., Lwin M.O. (2013). Cognitive, personality, and social factors associated with adolescents’ online personal information disclosure. J. Adolesc..

[24] Bryce J., Klang M. (2009). Young people, disclosure of personal information and online privacy: Control, choice and consequences. Inf. Secur. Tech. Rep..

[25] Bryce J., Fraser J. (2014). The role of disclosure of personal information in the evaluation of risk and trust in young peoples’ online interactions. Comput. Hum. Behav..

[26] EU General Data Protection Regulation. https://www.gdpreu.org/the-regulation/key-concepts/special-categories-personal-data/.

[27] Lin W.-Y., Zhang X., Song H., Omori K. (2016). Health information seeking in the Web 2.0 age: Trust in social media, uncertainty reduction, and self-disclosure. Comput. Hum. Behav..

[28] Su S., Pettit G.S., Erath S.A. (2016). Peer relations, parental social coaching, and young adolescent social anxiety. J. Appl. Dev. Psycho..

[29] Landoll R.R., La Greca A.M., Lai B.S., Chan S.F., Herge W.M. (2015). Cyber victimization by peers: Prospective associations with adolescent social anxiety and depressive symptoms. J. Adolesc..

[30] Vandebosch H., Van Cleemput K. (2009). Cyberbullying among youngsters: Profiles of bullies and victims. New Media Soc..

[31] Walrave M., Heirman W. (2010). Cyberbullying: Predicting victimisation and perpetration. Child. Soc..

[32] Chen Q.Q., Lo C.K., Zhu Y., Cheung A., Chan K.L., Ip P. (2018). Family poly-victimization and cyberbullying among adolescents in a Chinese school sample. Child Abuse Negl..

[33] McIntyre V. (2016). Do (x) you really want to hurt me: Adapting IIED as a solution to doxing by reshaping intent. Tul. J. Tech. Intell. Prop..

[34] Blessman S. Doxing and law enforcement: What to look for, how to prevent it. https://www.officer.com/investigations/article/12219040/doxing-and-law-enforcement-what-to-look-for-and-prevent.

[35] Ttofi M., Farrington D., Baldry A. (2008). Effectiveness of Programmes to Reduce School Bullying: A Systematic Review.

[36] Vaala S.E., Bleakley A. (2015). Monitoring, mediating, and modeling: Parental influence on adolescent computer and Internet use in the United States. J. Child. Media.

[37] Martínez I., Fuentes M., García F., Madrid I. (2013). The parenting style as protective or risk factor for substance use and other behavior problems among Spanish adolescents. Adicciones.

